# Crystal structure of diethyl 3,3′-[(4-nitro­phen­yl)methyl­ene]bis­(1*H*-indole-2-carboxyl­ate)

**DOI:** 10.1107/S2056989017016929

**Published:** 2017-11-28

**Authors:** Hong-Shun Sun, Yu-Long Li, Hong Jiang, Yu-Liang Chen, Ya-Di Hu

**Affiliations:** a Targeted MRI Contrast Agents Laboratory of Jiangsu Province, Nanjing Polytechnic Institute, Geguan Road No.265 Nanjing, Nanjing 210048, People’s Republic of China

**Keywords:** crystal structure, bis­indole, crystal structure, MRI, contrast agent

## Abstract

In the title compound, C_29_H_25_N_3_O_6_, the mean planes of the two indole ring systems (r.m.s. deviations = 0.0115 and 0.0082 Å) are approximately perpendic­ular to one another, making a dihedral angle of 89.7 (5)°; the benzene ring is twisted with respect to the two indole ring systems by 52.6 (4) and 88.2 (3)°. In the crystal, pairs of N—H⋯O hydrogen bonds link the mol­ecules into the inversion dimers, which are further linked into supra­molecular chains along the *b*-axis direction. Weak C—H⋯O hydrogen bonds and C—H⋯π inter­actions are also observed in the crystal.

## Abstract 2

In the title compound, the two indole ring systems are approximately perpendicular to one another, making a dihedral angle of 89.7 (5)°. In the crystal, pairs of N—H⋯O hydrogen bonds link the mol­ecules into the inversion dimers, which are further linked into supra­molecular chains.

## Chemical context   

Bis(indol­yl)methane derivatives are abundantly present in various terrestrial and marine natural resources (Porter *et al.*, 1977 ▸; Sundberg, 1996 ▸). They are important anti­biotics in the field of pharmaceuticals with diverse activities, such as anti­cancer, anti­leishmanial and anti­hyperlipidemic (Chang *et al.*, 1999 ▸; Ge *et al.*, 1999 ▸). On the other hand, bis­(indoly)methane derivatives can also be used as a precursor for MRI necrosis avid contrast agents (Ni, 2008 ▸). In recent years, we have reported the synthesis and crystal structures of some similar bis­(indoly)methane compounds (Sun *et al.*, 2012 ▸, 2015 ▸; Li *et al.*, 2014 ▸; Lu *et al.*, 2014 ▸). Now we report herein on the crystal structure of the title bis­(indoly)methane compound.
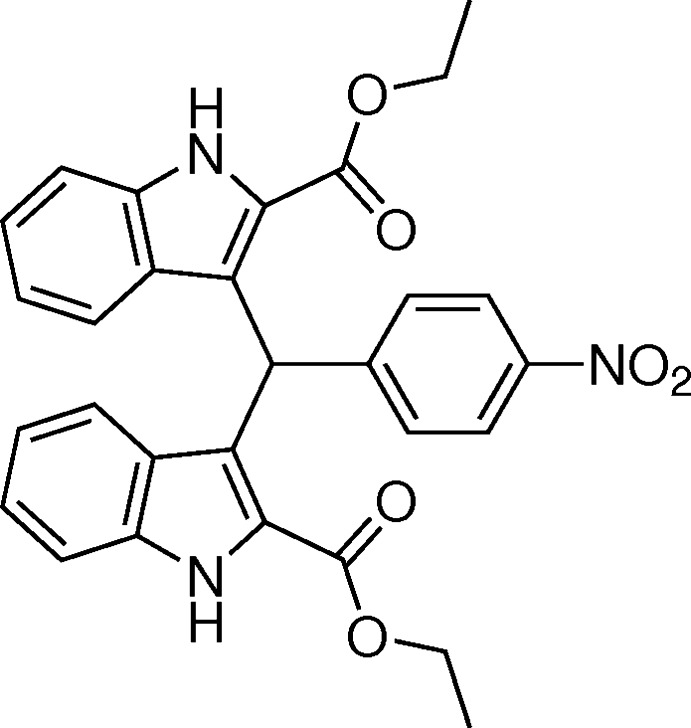


## Structural commentary   

**Figure 1:**
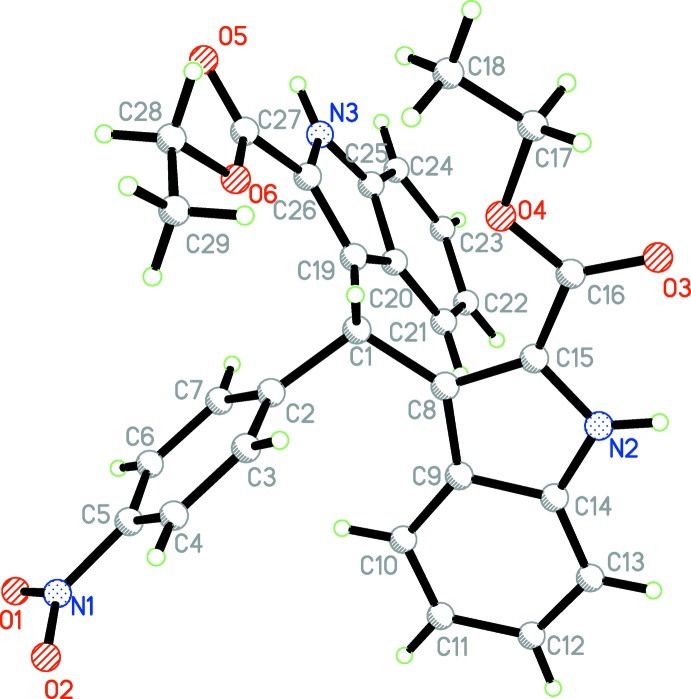
The mol­ecular structure of the title mol­ecule with the atom-labelling scheme. Displacement ellipsoids are drawn at the 30% probability level.

The mol­ecular structure of the title compound is shown in Fig. 1 ▸. The overall conformation of the mol­ecule is affected by intra­molecular C10—H10*A*⋯*Cg*3 and C21—H21*A*⋯*Cg*1 inter­actions (Table 1 ▸). The two indole ring systems are nearly perpendicular to one another [dihedral angle = 89.7 (5)°] while the benzene ring (C2–C7) is twisted to the N2/C8–C15 and N3/C19–C26 indole ring systems by dihedral angles of 52.6 (4) and 88.2 (3)°, respectively. The carboxyl groups are approximately coplanar with the attached indole ring systems, the dihedral angles between the carboxyl groups and the mean plane of the N2/C8–C15 and N3/C19–C26 indole ring systems are 12.5 (4) and 4.9 (5)°, respectively.

## Supra­molecular features   

**Figure 2:**
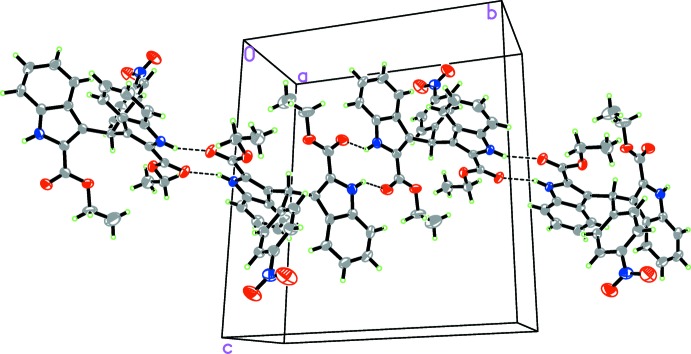
A packing diagram of the title compound. The N—H⋯O Hydrogen bonds are shown as dashed lines.

In the crystal, pairs of N2—H2*A*⋯O3^i^ and N3—H3*A*⋯O5^ii^ hydrogen bonds link the mol­ecules into the inversion dimers, which are further shown as supra­molecular chains propagating along the *b*-axis direction (Table 1 ▸ and Fig. 2 ▸). In the crystal, weak C—H⋯O hydrogen bonds and C—H⋯π inter­actions are also observed, linking the chains to form a three-dimensional supramolecular structure.

## Database survey   

Several similar structures have been reported previously, *i.e.* diethyl 3,3′-(phenyl­methyl­ene)bis­(1*H*-indole-2-carboxyl­ate) (Sun *et al.*, 2012 ▸) and dimethyl 3,3′-[(4-fluoro­phen­yl)methyl­ene]bis­(1*H*-indole-2-carboxyl­ate) (Sun *et al.*, 2015 ▸) and dimethyl 3,3′-[(4-chloro­phen­yl) methyl­ene]bis­(1*H*-indole-2-carboxyl­ate) (Li *et al.*, 2014 ▸) and dimethyl 3,3′-[(3-fluoro­phen­yl)methyl­ene]bis­(1*H*-indole-2-carboxyl­ate) (Lu *et al.*, 2014 ▸). In these structures, the two indole ring systems are also nearly perpendicular to one another, making dihedral angles of 82.0 (5), 84.0 (5), 79.5 (4) and 87.8 (5)°, respectively.

## Synthesis and crystallization   

Ethyl indole-2-carboxyl­ate (1.88 g, 10 mmol) was dissolved in 20 ml ethanol; commercially available 4-nitro­benzaldehyde (0.76 g, 5 mmol) was added and the mixture was heated to reflux temperature. Concentrated HCl (0.5 ml) was added and the reaction was left for 1 h. After cooling, the yellow product was filtered off and washed thoroughly with ethanol. The reaction was monitored with TLC (AcOEt:hexane = 1:3). Single crystals of the title compound suitable for X-ray analysis were obtained by slow evaporation of a methanol solution (yield 93%).

## Refinement   

Crystal data, data collection and structure refinement details are summarized in Table 2 ▸. H atoms were positioned geometrically with N—H = 0.86 Å and C—H = 0.93–0.98 Å, and constrained to ride on their parent atoms with *U*_iso_(H) = *xU*_eq_(C,N), where *x* = 1.5 for methyl H atoms and 1.2 for other H atoms.

## Supplementary Material

Crystal structure: contains datablock(s) I, global. DOI: 10.1107/S2056989017016929/xu5911sup1.cif

Structure factors: contains datablock(s) I. DOI: 10.1107/S2056989017016929/xu5911Isup2.hkl

Supporting information file. DOI: 10.1107/S2056989017016929/xu5911Isup3.cml

CCDC reference: 1587329

Additional supporting information:  crystallographic information; 3D view; checkCIF report

## Figures and Tables

**Table 1:** Hydrogen-bond geometry (Å, °) *Cg*1, *Cg*3 and *Cg*5 are the centroids of the N2/C8/C9/C14/C15 pyrrole, C2–C7 benzene and C21–C26 benzene rings, respectively.

*D*—H⋯*A*	*D*—H	H⋯*A*	*D*⋯*A*	*D*—H⋯*A*
N2—H2*A*⋯O3^i^	0.86	2.30	3.003 (3)	139
N3—H3*A*⋯O5^ii^	0.86	2.14	2.956 (3)	158
C11—H11*A*⋯O5^iii^	0.93	2.58	3.501 (4)	171
C17—H17*B*⋯O1^iv^	0.97	2.58	3.261 (5)	128
C29—H29*A*⋯O1^v^	0.96	2.51	3.281 (4)	137
C10—H10*A*⋯*Cg*3	0.93	2.69	3.431 (3)	138
C21—H21*A*⋯*Cg*1	0.93	2.88	3.570 (3)	132
C28—H28*A*⋯*Cg*5^vi^	0.97	2.85	3.718 (3)	150

Symmetry codes: (i) 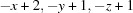; (ii) 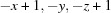; (iii) 
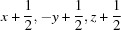
; (iv) 
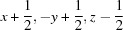
; (v) 
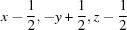
; (vi) 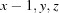.

**Table 2:** Experimental details

Crystal data	
Chemical formula	C_29_H_25_N_3_O_6_
*M*_r_	511.52
Crystal system, space group	Monoclinic, *P*2_1_/*n*
Temperature (K)	293
*a*, *b*, *c* (Å)	8.8040 (18), 15.804 (3), 18.266 (4)
β (°)	98.78 (3)
*V* (Å^3^)	2511.7 (9)
*Z*	4
Radiation type	Mo *K*α
μ (mm^−1^)	0.10
Crystal size (mm)	0.30 × 0.20 × 0.10

Data collection	
Diffractometer	Nonius CAD-4
Absorption correction	ψ scan (North *et al.*, 1968 ▸)
*T*_min_, *T*_max_	0.972, 0.991
No. of measured, independent and observed [*I* > 2σ(*I*)] reflections	4925, 4611, 2963
*R*_int_	0.043
(sin θ/λ)_max_ (Å^−1^)	0.603

Refinement	
*R*[*F* ^2^ > 2σ(*F* ^2^)], *wR*(*F* ^2^), *S*	0.059, 0.164, 1.00
No. of reflections	4611
No. of parameters	343
H-atom treatment	H-atom parameters constrained
Δρ_max_, Δρ_min_ (e Å^−3^)	0.39, −0.28

Computer programs: *CAD-4 EXPRESS* (Enraf–Nonius, 1994 ▸), *XCAD4* (Harms & Wocadlo, 1995 ▸) and *SHELXTL* (Sheldrick, 2008 ▸).

## supplementary crystallographic information

### Crystal data

**Table:**

C_29_H_25_N_3_O_6_	*F*(000) = 1072
*M**_r_* = 511.52	*D*_x_ = 1.353 Mg m^−^^3^
Monoclinic, *P*2_1_/*n*	Mo *K*α radiation, λ = 0.71073 Å
Hall symbol: -P 2yn	Cell parameters from 25 reflections
*a* = 8.8040 (18) Å	θ = 9–13°
*b* = 15.804 (3) Å	µ = 0.10 mm^−^^1^
*c* = 18.266 (4) Å	*T* = 293 K
β = 98.78 (3)°	Block, colorless
*V* = 2511.7 (9) Å^3^	0.30 × 0.20 × 0.10 mm
*Z* = 4	

### Data collection

**Table:**

Nonius CAD-4 diffractometer	2963 reflections with *I* > 2σ(*I*)
Radiation source: fine-focus sealed tube	*R*_int_ = 0.043
Graphite monochromator	θ_max_ = 25.4°, θ_min_ = 1.7°
ω/2θ scans	*h* = 0→10
Absorption correction: ψ scan (North *et al.*, 1968)	*k* = 0→19
*T*_min_ = 0.972, *T*_max_ = 0.991	*l* = −22→22
4925 measured reflections	3 standard reflections every 200 reflections
4611 independent reflections	intensity decay: 1%

### Refinement

**Table:**

Refinement on *F*^2^	Primary atom site location: structure-invariant direct methods
Least-squares matrix: full	Secondary atom site location: difference Fourier map
*R*[*F*^2^ > 2σ(*F*^2^)] = 0.059	Hydrogen site location: inferred from neighbouring sites
*wR*(*F*^2^) = 0.164	H-atom parameters constrained
*S* = 1.00	*w* = 1/[σ^2^(*F*_o_^2^) + (0.098*P*)^2^] where *P* = (*F*_o_^2^ + 2*F*_c_^2^)/3
4611 reflections	(Δ/σ)_max_ < 0.001
343 parameters	Δρ_max_ = 0.39 e Å^−^^3^
0 restraints	Δρ_min_ = −0.28 e Å^−^^3^

### Special details

**Table:**

Geometry. All esds (except the esd in the dihedral angle between two l.s. planes) are estimated using the full covariance matrix. The cell esds are taken into account individually in the estimation of esds in distances, angles and torsion angles; correlations between esds in cell parameters are only used when they are defined by crystal symmetry. An approximate (isotropic) treatment of cell esds is used for estimating esds involving l.s. planes.
Refinement. Refinement of F^2^ against ALL reflections. The weighted R-factor wR and goodness of fit S are based on F^2^, conventional R-factors R are based on F, with F set to zero for negative F^2^. The threshold expression of F^2^ > 2sigma(F^2^) is used only for calculating R-factors(gt) etc. and is not relevant to the choice of reflections for refinement. R-factors based on F^2^ are statistically about twice as large as those based on F, and R- factors based on ALL data will be even larger.

### Fractional atomic coordinates and isotropic or equivalent isotropic displacement parameters (Å^2^)

**Table:**

	*x*	*y*	*z*	*U*_iso_*/*U*_eq_	
N1	0.3019 (3)	0.33359 (18)	0.86721 (14)	0.0648 (7)	
C1	0.5958 (3)	0.31146 (14)	0.60845 (12)	0.0395 (5)	
H1A	0.5204	0.3259	0.5650	0.047*	
O1	0.3145 (3)	0.27719 (17)	0.91181 (14)	0.1013 (9)	
N2	0.8861 (2)	0.47555 (13)	0.58612 (12)	0.0517 (6)	
H2A	0.9392	0.5055	0.5600	0.062*	
C2	0.5083 (3)	0.31093 (14)	0.67433 (13)	0.0394 (6)	
O2	0.2403 (4)	0.39980 (19)	0.87776 (15)	0.1199 (11)	
N3	0.6690 (2)	0.09119 (12)	0.55132 (10)	0.0435 (5)	
H3A	0.6407	0.0442	0.5296	0.052*	
O3	0.8106 (2)	0.45055 (14)	0.43699 (10)	0.0729 (6)	
C3	0.4078 (3)	0.37605 (17)	0.68231 (14)	0.0546 (7)	
H3B	0.3864	0.4157	0.6445	0.065*	
O4	0.6050 (2)	0.37421 (13)	0.45415 (9)	0.0626 (5)	
C4	0.3383 (3)	0.38418 (18)	0.74445 (15)	0.0590 (7)	
H4A	0.2725	0.4291	0.7494	0.071*	
O5	0.3605 (2)	0.08873 (11)	0.49279 (11)	0.0597 (5)	
C5	0.3687 (3)	0.32430 (16)	0.79870 (14)	0.0485 (6)	
O6	0.33771 (19)	0.22355 (10)	0.52526 (10)	0.0544 (5)	
C6	0.4600 (3)	0.25512 (16)	0.79184 (14)	0.0488 (6)	
H6A	0.4746	0.2137	0.8284	0.059*	
C7	0.5301 (3)	0.24868 (15)	0.72855 (13)	0.0453 (6)	
H7A	0.5920	0.2023	0.7226	0.054*	
C8	0.7142 (3)	0.38043 (14)	0.61580 (13)	0.0413 (6)	
C9	0.7960 (3)	0.41747 (15)	0.68231 (14)	0.0452 (6)	
C10	0.7929 (3)	0.40819 (18)	0.75905 (14)	0.0569 (7)	
H10A	0.7264	0.3696	0.7760	0.068*	
C11	0.8885 (3)	0.45645 (19)	0.80769 (16)	0.0653 (8)	
H11A	0.8854	0.4506	0.8581	0.078*	
C12	0.9902 (3)	0.51407 (18)	0.78475 (16)	0.0610 (8)	
H12A	1.0526	0.5464	0.8198	0.073*	
C13	0.9999 (3)	0.52399 (16)	0.71204 (16)	0.0553 (7)	
H13A	1.0699	0.5616	0.6966	0.066*	
C14	0.9023 (3)	0.47627 (15)	0.66117 (14)	0.0479 (6)	
C15	0.7728 (3)	0.42011 (15)	0.55827 (13)	0.0437 (6)	
C16	0.7342 (3)	0.41604 (17)	0.47798 (15)	0.0518 (7)	
C17	0.5564 (4)	0.3709 (3)	0.37461 (16)	0.0888 (11)	
H17A	0.5529	0.4278	0.3545	0.107*	
H17B	0.6304	0.3386	0.3519	0.107*	
C18	0.4103 (5)	0.3332 (3)	0.3575 (2)	0.1267 (17)	
H18A	0.3794	0.3330	0.3048	0.190*	
H18B	0.3372	0.3649	0.3804	0.190*	
H18C	0.4147	0.2761	0.3757	0.190*	
C19	0.6591 (3)	0.22443 (14)	0.59271 (12)	0.0384 (5)	
C20	0.8111 (3)	0.19214 (15)	0.61572 (13)	0.0431 (6)	
C21	0.9467 (3)	0.22243 (18)	0.65751 (15)	0.0549 (7)	
H21A	0.9506	0.2768	0.6771	0.066*	
C22	1.0729 (3)	0.1716 (2)	0.66927 (17)	0.0656 (8)	
H22A	1.1630	0.1920	0.6968	0.079*	
C23	1.0702 (3)	0.0891 (2)	0.64087 (18)	0.0699 (9)	
H23A	1.1584	0.0560	0.6499	0.084*	
C24	0.9407 (3)	0.05644 (18)	0.60030 (16)	0.0593 (7)	
H24A	0.9387	0.0017	0.5816	0.071*	
C25	0.8115 (3)	0.10832 (16)	0.58803 (13)	0.0444 (6)	
C26	0.5775 (3)	0.16072 (15)	0.55434 (12)	0.0414 (6)	
C27	0.4159 (3)	0.15280 (15)	0.52078 (13)	0.0415 (6)	
C28	0.1776 (3)	0.22520 (18)	0.49292 (16)	0.0582 (7)	
H28A	0.1201	0.1837	0.5166	0.070*	
H28B	0.1661	0.2126	0.4404	0.070*	
C29	0.1216 (4)	0.3115 (2)	0.5050 (2)	0.0800 (10)	
H29A	0.0149	0.3156	0.4843	0.120*	
H29B	0.1797	0.3518	0.4813	0.120*	
H29C	0.1340	0.3230	0.5571	0.120*	

### Atomic displacement parameters (Å^2^)

**Table:**

	*U*^11^	*U*^22^	*U*^33^	*U*^12^	*U*^13^	*U*^23^
N1	0.0613 (15)	0.0828 (19)	0.0541 (15)	0.0045 (14)	0.0213 (12)	−0.0012 (14)
C1	0.0445 (13)	0.0358 (12)	0.0368 (13)	−0.0016 (11)	0.0023 (10)	−0.0019 (10)
O1	0.131 (2)	0.110 (2)	0.0739 (16)	0.0141 (17)	0.0519 (16)	0.0261 (15)
N2	0.0568 (13)	0.0472 (12)	0.0531 (14)	−0.0065 (11)	0.0147 (11)	0.0026 (10)
C2	0.0426 (13)	0.0351 (12)	0.0395 (13)	−0.0071 (10)	0.0026 (11)	−0.0042 (10)
O2	0.168 (3)	0.117 (2)	0.0915 (19)	0.057 (2)	0.0745 (19)	0.0109 (16)
N3	0.0516 (13)	0.0353 (11)	0.0438 (12)	−0.0017 (9)	0.0073 (10)	−0.0073 (9)
O3	0.0749 (14)	0.0936 (16)	0.0510 (12)	−0.0223 (12)	0.0125 (10)	0.0160 (11)
C3	0.0665 (18)	0.0512 (15)	0.0460 (16)	0.0097 (14)	0.0085 (13)	0.0022 (12)
O4	0.0659 (13)	0.0848 (14)	0.0364 (10)	−0.0177 (11)	0.0052 (9)	0.0009 (9)
C4	0.0647 (18)	0.0619 (17)	0.0528 (17)	0.0138 (15)	0.0168 (14)	−0.0025 (14)
O5	0.0555 (11)	0.0509 (11)	0.0695 (13)	−0.0054 (9)	−0.0010 (9)	−0.0194 (9)
C5	0.0477 (15)	0.0562 (15)	0.0428 (14)	−0.0033 (13)	0.0108 (12)	−0.0044 (12)
O6	0.0485 (11)	0.0417 (10)	0.0691 (13)	0.0012 (8)	−0.0038 (9)	−0.0040 (9)
C6	0.0537 (16)	0.0494 (14)	0.0436 (15)	−0.0069 (13)	0.0080 (12)	0.0046 (12)
C7	0.0471 (14)	0.0408 (13)	0.0480 (15)	0.0002 (11)	0.0077 (11)	−0.0013 (11)
C8	0.0485 (14)	0.0363 (12)	0.0395 (13)	−0.0006 (11)	0.0078 (11)	−0.0013 (10)
C9	0.0521 (15)	0.0362 (13)	0.0477 (15)	−0.0031 (11)	0.0093 (12)	−0.0071 (11)
C10	0.0652 (18)	0.0632 (17)	0.0424 (15)	−0.0160 (14)	0.0089 (13)	−0.0068 (13)
C11	0.0708 (19)	0.077 (2)	0.0483 (16)	−0.0150 (17)	0.0078 (14)	−0.0159 (15)
C12	0.0602 (18)	0.0591 (17)	0.0608 (19)	−0.0081 (14)	0.0005 (14)	−0.0220 (14)
C13	0.0510 (16)	0.0396 (14)	0.075 (2)	−0.0094 (12)	0.0088 (14)	−0.0102 (13)
C14	0.0500 (15)	0.0401 (13)	0.0537 (16)	0.0002 (12)	0.0079 (12)	−0.0026 (11)
C15	0.0494 (14)	0.0417 (13)	0.0406 (14)	−0.0012 (12)	0.0084 (11)	0.0007 (11)
C16	0.0536 (16)	0.0558 (16)	0.0466 (15)	−0.0012 (13)	0.0093 (13)	0.0070 (13)
C17	0.087 (2)	0.135 (3)	0.0402 (17)	−0.019 (2)	−0.0032 (16)	0.0002 (19)
C18	0.105 (3)	0.185 (5)	0.088 (3)	−0.040 (3)	0.004 (3)	−0.023 (3)
C19	0.0450 (14)	0.0379 (12)	0.0326 (12)	−0.0029 (11)	0.0066 (10)	−0.0029 (10)
C20	0.0454 (14)	0.0488 (14)	0.0355 (13)	0.0005 (12)	0.0073 (11)	0.0003 (11)
C21	0.0520 (16)	0.0590 (16)	0.0514 (16)	−0.0032 (14)	0.0003 (13)	−0.0079 (13)
C22	0.0505 (17)	0.075 (2)	0.068 (2)	−0.0010 (16)	−0.0007 (14)	−0.0014 (16)
C23	0.0517 (18)	0.076 (2)	0.080 (2)	0.0135 (16)	0.0053 (16)	0.0071 (17)
C24	0.0564 (17)	0.0507 (16)	0.0708 (19)	0.0089 (14)	0.0100 (15)	−0.0001 (14)
C25	0.0457 (14)	0.0463 (14)	0.0417 (14)	−0.0009 (12)	0.0081 (11)	0.0025 (11)
C26	0.0454 (14)	0.0472 (14)	0.0316 (12)	−0.0009 (11)	0.0060 (10)	0.0010 (10)
C27	0.0509 (15)	0.0385 (13)	0.0348 (13)	−0.0035 (12)	0.0057 (11)	−0.0028 (10)
C28	0.0465 (16)	0.0606 (17)	0.0643 (18)	−0.0006 (13)	−0.0016 (13)	−0.0003 (14)
C29	0.070 (2)	0.068 (2)	0.099 (3)	0.0182 (17)	0.0043 (19)	0.0002 (18)

### Geometric parameters (Å, º)

**Table:**

N1—O1	1.201 (3)	C10—H10A	0.9300
N1—O2	1.208 (3)	C11—C12	1.386 (4)
N1—C5	1.469 (3)	C11—H11A	0.9300
C1—C8	1.500 (3)	C12—C13	1.353 (4)
C1—C2	1.524 (3)	C12—H12A	0.9300
C1—C19	1.528 (3)	C13—C14	1.388 (4)
C1—H1A	0.9800	C13—H13A	0.9300
N2—C14	1.356 (3)	C15—C16	1.455 (3)
N2—C15	1.366 (3)	C17—C18	1.408 (5)
N2—H2A	0.8600	C17—H17A	0.9700
C2—C3	1.380 (3)	C17—H17B	0.9700
C2—C7	1.388 (3)	C18—H18A	0.9600
N3—C25	1.356 (3)	C18—H18B	0.9600
N3—C26	1.369 (3)	C18—H18C	0.9600
N3—H3A	0.8600	C19—C26	1.367 (3)
O3—C16	1.210 (3)	C19—C20	1.434 (3)
C3—C4	1.375 (3)	C20—C21	1.399 (3)
C3—H3B	0.9300	C20—C25	1.418 (3)
O4—C16	1.330 (3)	C21—C22	1.361 (4)
O4—C17	1.452 (3)	C21—H21A	0.9300
C4—C5	1.367 (4)	C22—C23	1.402 (4)
C4—H4A	0.9300	C22—H22A	0.9300
O5—C27	1.204 (3)	C23—C24	1.363 (4)
C5—C6	1.374 (3)	C23—H23A	0.9300
O6—C27	1.322 (3)	C24—C25	1.392 (4)
O6—C28	1.443 (3)	C24—H24A	0.9300
C6—C7	1.395 (3)	C26—C27	1.465 (3)
C6—H6A	0.9300	C28—C29	1.478 (4)
C7—H7A	0.9300	C28—H28A	0.9700
C8—C15	1.389 (3)	C28—H28B	0.9700
C8—C9	1.439 (3)	C29—H29A	0.9600
C9—C14	1.414 (3)	C29—H29B	0.9600
C9—C10	1.414 (3)	C29—H29C	0.9600
C10—C11	1.360 (4)		
			
O1—N1—O2	122.1 (3)	N2—C15—C16	116.7 (2)
O1—N1—C5	119.7 (3)	C8—C15—C16	133.3 (2)
O2—N1—C5	118.1 (3)	O3—C16—O4	123.4 (3)
C8—C1—C2	111.17 (18)	O3—C16—C15	122.5 (3)
C8—C1—C19	113.74 (19)	O4—C16—C15	114.1 (2)
C2—C1—C19	112.73 (18)	C18—C17—O4	110.9 (3)
C8—C1—H1A	106.2	C18—C17—H17A	109.5
C2—C1—H1A	106.2	O4—C17—H17A	109.5
C19—C1—H1A	106.2	C18—C17—H17B	109.5
C14—N2—C15	109.9 (2)	O4—C17—H17B	109.5
C14—N2—H2A	125.1	H17A—C17—H17B	108.0
C15—N2—H2A	125.1	C17—C18—H18A	109.5
C3—C2—C7	118.3 (2)	C17—C18—H18B	109.5
C3—C2—C1	119.3 (2)	H18A—C18—H18B	109.5
C7—C2—C1	122.4 (2)	C17—C18—H18C	109.5
C25—N3—C26	109.01 (19)	H18A—C18—H18C	109.5
C25—N3—H3A	125.5	H18B—C18—H18C	109.5
C26—N3—H3A	125.5	C26—C19—C20	106.4 (2)
C4—C3—C2	122.0 (2)	C26—C19—C1	125.5 (2)
C4—C3—H3B	119.0	C20—C19—C1	128.0 (2)
C2—C3—H3B	119.0	C21—C20—C25	117.8 (2)
C16—O4—C17	116.9 (2)	C21—C20—C19	135.9 (2)
C5—C4—C3	118.1 (3)	C25—C20—C19	106.3 (2)
C5—C4—H4A	121.0	C22—C21—C20	119.4 (3)
C3—C4—H4A	121.0	C22—C21—H21A	120.3
C4—C5—C6	122.5 (2)	C20—C21—H21A	120.3
C4—C5—N1	119.2 (2)	C21—C22—C23	121.6 (3)
C6—C5—N1	118.2 (2)	C21—C22—H22A	119.2
C27—O6—C28	118.4 (2)	C23—C22—H22A	119.2
C5—C6—C7	118.2 (2)	C24—C23—C22	121.3 (3)
C5—C6—H6A	120.9	C24—C23—H23A	119.4
C7—C6—H6A	120.9	C22—C23—H23A	119.4
C2—C7—C6	120.6 (2)	C23—C24—C25	117.4 (3)
C2—C7—H7A	119.7	C23—C24—H24A	121.3
C6—C7—H7A	119.7	C25—C24—H24A	121.3
C15—C8—C9	105.0 (2)	N3—C25—C24	129.3 (2)
C15—C8—C1	126.4 (2)	N3—C25—C20	108.1 (2)
C9—C8—C1	128.5 (2)	C24—C25—C20	122.6 (2)
C14—C9—C10	116.9 (2)	C19—C26—N3	110.2 (2)
C14—C9—C8	107.7 (2)	C19—C26—C27	132.7 (2)
C10—C9—C8	135.4 (2)	N3—C26—C27	117.0 (2)
C11—C10—C9	119.0 (3)	O5—C27—O6	123.8 (2)
C11—C10—H10A	120.5	O5—C27—C26	123.7 (2)
C9—C10—H10A	120.5	O6—C27—C26	112.5 (2)
C10—C11—C12	122.3 (3)	O6—C28—C29	106.4 (2)
C10—C11—H11A	118.8	O6—C28—H28A	110.4
C12—C11—H11A	118.8	C29—C28—H28A	110.4
C13—C12—C11	120.9 (3)	O6—C28—H28B	110.4
C13—C12—H12A	119.5	C29—C28—H28B	110.4
C11—C12—H12A	119.5	H28A—C28—H28B	108.6
C12—C13—C14	117.9 (3)	C28—C29—H29A	109.5
C12—C13—H13A	121.0	C28—C29—H29B	109.5
C14—C13—H13A	121.0	H29A—C29—H29B	109.5
N2—C14—C13	129.8 (2)	C28—C29—H29C	109.5
N2—C14—C9	107.4 (2)	H29A—C29—H29C	109.5
C13—C14—C9	122.8 (2)	H29B—C29—H29C	109.5
N2—C15—C8	110.0 (2)		
			
C8—C1—C2—C3	69.9 (3)	C9—C8—C15—C16	−175.6 (3)
C19—C1—C2—C3	−161.1 (2)	C1—C8—C15—C16	6.7 (4)
C8—C1—C2—C7	−107.7 (2)	C17—O4—C16—O3	0.0 (4)
C19—C1—C2—C7	21.4 (3)	C17—O4—C16—C15	177.5 (3)
C7—C2—C3—C4	4.9 (4)	N2—C15—C16—O3	9.9 (4)
C1—C2—C3—C4	−172.8 (2)	C8—C15—C16—O3	−172.8 (3)
C2—C3—C4—C5	−1.4 (4)	N2—C15—C16—O4	−167.6 (2)
C3—C4—C5—C6	−2.9 (4)	C8—C15—C16—O4	9.8 (4)
C3—C4—C5—N1	177.1 (2)	C16—O4—C17—C18	−173.4 (3)
O1—N1—C5—C4	172.3 (3)	C8—C1—C19—C26	−150.9 (2)
O2—N1—C5—C4	−10.4 (4)	C2—C1—C19—C26	81.3 (3)
O1—N1—C5—C6	−7.7 (4)	C8—C1—C19—C20	30.6 (3)
O2—N1—C5—C6	169.7 (3)	C2—C1—C19—C20	−97.1 (3)
C4—C5—C6—C7	3.4 (4)	C26—C19—C20—C21	−177.8 (3)
N1—C5—C6—C7	−176.6 (2)	C1—C19—C20—C21	0.9 (4)
C3—C2—C7—C6	−4.3 (4)	C26—C19—C20—C25	0.6 (2)
C1—C2—C7—C6	173.3 (2)	C1—C19—C20—C25	179.3 (2)
C5—C6—C7—C2	0.3 (4)	C25—C20—C21—C22	0.7 (4)
C2—C1—C8—C15	−154.3 (2)	C19—C20—C21—C22	179.0 (3)
C19—C1—C8—C15	77.2 (3)	C20—C21—C22—C23	−0.4 (4)
C2—C1—C8—C9	28.5 (3)	C21—C22—C23—C24	−0.1 (5)
C19—C1—C8—C9	−100.0 (3)	C22—C23—C24—C25	0.2 (4)
C15—C8—C9—C14	−1.3 (3)	C26—N3—C25—C24	178.9 (2)
C1—C8—C9—C14	176.3 (2)	C26—N3—C25—C20	0.1 (3)
C15—C8—C9—C10	178.7 (3)	C23—C24—C25—N3	−178.5 (3)
C1—C8—C9—C10	−3.7 (5)	C23—C24—C25—C20	0.2 (4)
C14—C9—C10—C11	1.1 (4)	C21—C20—C25—N3	178.3 (2)
C8—C9—C10—C11	−178.8 (3)	C19—C20—C25—N3	−0.4 (3)
C9—C10—C11—C12	−0.6 (4)	C21—C20—C25—C24	−0.6 (4)
C10—C11—C12—C13	−0.8 (5)	C19—C20—C25—C24	−179.4 (2)
C11—C12—C13—C14	1.6 (4)	C20—C19—C26—N3	−0.6 (3)
C15—N2—C14—C13	−178.3 (2)	C1—C19—C26—N3	−179.29 (19)
C15—N2—C14—C9	1.0 (3)	C20—C19—C26—C27	176.1 (2)
C12—C13—C14—N2	178.2 (3)	C1—C19—C26—C27	−2.6 (4)
C12—C13—C14—C9	−1.0 (4)	C25—N3—C26—C19	0.3 (3)
C10—C9—C14—N2	−179.8 (2)	C25—N3—C26—C27	−176.9 (2)
C8—C9—C14—N2	0.2 (3)	C28—O6—C27—O5	−1.9 (4)
C10—C9—C14—C13	−0.4 (4)	C28—O6—C27—C26	178.7 (2)
C8—C9—C14—C13	179.6 (2)	C19—C26—C27—O5	−174.8 (3)
C14—N2—C15—C8	−1.9 (3)	N3—C26—C27—O5	1.7 (3)
C14—N2—C15—C16	176.1 (2)	C19—C26—C27—O6	4.6 (4)
C9—C8—C15—N2	1.9 (3)	N3—C26—C27—O6	−178.84 (19)
C1—C8—C15—N2	−175.8 (2)	C27—O6—C28—C29	−179.0 (2)

### Hydrogen-bond geometry (Å, º)

Cg1, Cg3 and Cg5 are the centroids of the N2/C8/C9/C14/C15 pyrrole, C2–C7 benzene and C21–C26 benzene rings, respectively.

**Table:**

*D*—H···*A*	*D*—H	H···*A*	*D*···*A*	*D*—H···*A*
N2—H2*A*···O3^i^	0.86	2.30	3.003 (3)	139
N3—H3*A*···O5^ii^	0.86	2.14	2.956 (3)	158
C11—H11*A*···O5^iii^	0.93	2.58	3.501 (4)	171
C17—H17*B*···O1^iv^	0.97	2.58	3.261 (5)	128
C29—H29*A*···O1^v^	0.96	2.51	3.281 (4)	137
C10—H10*A*···*Cg*3	0.93	2.69	3.431 (3)	138
C21—H21*A*···*Cg*1	0.93	2.88	3.570 (3)	132
C28—H28*A*···*Cg*5^vi^	0.97	2.85	3.718 (3)	150

Symmetry codes: (i) −*x*+2, −*y*+1, −*z*+1; (ii) −*x*+1, −*y*, −*z*+1; (iii) *x*+1/2, −*y*+1/2, *z*+1/2; (iv) *x*+1/2, −*y*+1/2, *z*−1/2; (v) *x*−1/2, −*y*+1/2, *z*−1/2; (vi) *x*−1, *y*, *z*.
